# Psychometric properties of the experiences of maternity care scale among Iranian women

**DOI:** 10.1186/s12913-024-11065-1

**Published:** 2024-05-11

**Authors:** Elham Jafari, Mohammad Asghari-Jafarabadi, Mojgan Mirghafourvand, Sakineh Mohammad-Alizadeh-Charandabi

**Affiliations:** 1 Student Research Comittee, Department of Midwifery, Faculty of Nursing and Midwifery, Tabriz University of Medical Sciences, Tabriz, Iran; 2 Cabrini Research, Cabrini Health, Malvern, VIC 3144 Australia; 3 School of Public Health and Preventive Medicine, Monash University, Melbourne, VIC 3004 Australia; 4 Department of Psychiatry, School of Clinical Sciences, Monash University, Clayton, VIC, 3168 Australia; 5 Social Determinants of Health Research Center, Tabriz University of Medical Sciences, Tabriz, Iran; 6 Department of Midwifery, Faculty of Nursing and Midwifery, Tabriz University of Medical Sciences, Shariati Ave, P.O. Box: 51745- 347, Tabriz, 513897977 Iran

**Keywords:** Women, Psychometric, Maternal health, Perinatal care, Postnatal care, Pregnancy, Labour, Care quality

## Abstract

**Background:**

Assessing women’s perceptions of the care they receive is crucial for evaluating the quality of maternity care. Women’s perceptions are influenced by the care received during pregnancy, labour and birth, and the postpartum period, each of which with unique conditions, expectations, and requirements. In England, three Experience of Maternity Care (EMC) scales – Pregnancy, Labour and Birth, and Postnatal – have been developed to assess women’s experiences from pregnancy through the postpartum period. This study aimed to validate these scales within the Iranian context.

**Methods:**

A methodological cross-sectional study was conducted from December 2022 to August 2023 at selected health centers in Tabriz, Iran. A panel of 16 experts assessed the qualitative and quantitative content validity of the scales and 10 women assessed the face validity. A total of 540 eligible women, 1–6 months postpartum, participated in the study, with data from 216 women being used for exploratory factor analysis (EFA) and 324 women for confirmatory factor analysis (CFA) and other analyses. The Childbirth Experience Questionnaire-2 was employed to assess the convergent validity of the Labour and Birth Scale, whereas women’s age was used to assess the divergent validity of the scales. Test-retest reliability and internal consistency were also examined.

**Results:**

All items obtained an impact score above 1.5, with Content Validity Ratio and Content Validity Index exceeding 0.8. EFA demonstrated an excellent fit with the data (all Kaiser-Meyer-Olkin measures > 0.80, and all Bartlett’s *p* < 0.001). The Pregnancy Scale exhibited a five-factor structure, the Labour and Birth Scale a two-factor structure, and the Postnatal Scale a three-factor structure, explaining 66%, 57%, and 62% of the cumulative variance, respectively, for each scale. CFA indicated an acceptable fit with RMSEA ≤ 0.08, CFI ≥ 0.92, and NNFI ≥ 0.90. A significant correlation was observed between the Labour and Birth scale and the Childbirth Experience Questionnaire-2 (*r* = 0.82, *P* < 0.001). No significant correlation was found between the scales and women’s age. All three scales demonstrated good internal consistency (all Cronbach’s alpha values > 0.9) and test-retest reliability (all interclass correlation coefficient values > 0.8).

**Conclusions:**

The Persian versions of all three EMC scales exhibit robust psychometric properties for evaluating maternity care experiences among urban Iranian women. These scales can be utilized to assess the quality of current care, investigate the impact of different care models in various studies, and contribute to maternal health promotion programs and policies.

**Supplementary Information:**

The online version contains supplementary material available at 10.1186/s12913-024-11065-1.

## Background

Women’s experiences in maternity care are of paramount importance. Positive experiences have been associated with improved maternal and infant outcomes [1], while negative experiences have been associated with a higher risk of adverse postpartum mental health outcomes, including depression, anxiety, and fear of childbirth [2]. Women who have negative childbirth experiences may also postpone future pregnancies or choose not to conceive again [3]. Additionally, they often prefer cesarean section in their subsequent deliveries [4].

Improving maternal care begins with evaluating the current state of affairs from the perspective of women, who are the primary users of the system [5]. Assessing women’s maternity care experiences is crucial for evaluating the quality of care, as the findings can significantly impact policy decisions regarding the allocation of limited maternal care resources [6]. Recognizing this importance has led to the development of psychometric scales in this field [7].

The process of shaping women’s maternity care experiences starts during pregnancy and evolves through labour, birth, and the postpartum period [6]. However, existing scales primarily focus on assessing labour and birth experiences, with fewer tools available for evaluating pregnancy and postpartum experiences [8]. Recent efforts have aimed to develop scales that cover the entire maternity care continuum, such as the Pregnancy and Maternity Care Patients’ Experiences Questionnaire (PreMaPEQ) [9], Women’s Perception of Their Entire Maternity-care Experience [10], and Women’s Experience of Maternity Care (ESEM) [11].

While these scales address various aspects of maternity care, challenges hinder their effective use [7]. Some scales have an excessive number of items [9] or lack test-retest reliability [12]. Additionally, key statistical indices such as exploratory factor analysis (EFA) or confirmatory factor analysis (CFA) indices are not reported for some of them [9–11]. Another significant criticism is the inadequate reporting of content validity indicators [9–11]. It is crucial to consider women’s perspectives, needs, and expectations when assessing their experiences [8]. The lack of information on the content validity of the scales indicates a deficiency in women’s involvement in their design [7]. A recent systematic review of the psychometric properties of patient-reported measures of maternity care has also emphasized the importance of including women’s participation in the design of new scales, particularly for identifying relevant, understandable, and comprehensive items. The review also highlights the necessity of establishing the content validity of existing tools in other target populations [8].

In England, Redshaw and colleagues (2019) developed and validated three concise, related scales to assess women’s experiences of maternity care (EMC) during pregnancy (EMC-PR), labour and birth (EMC-LB), and early postnatal (EMC-PN). These scales were developed using psychological and social theories, and findings of qualitative interviews with women and previous surveys to effectively address the key needs of women. The simple and short design of the scales makes them practical for use, with initial testing demonstrating suitable factor scores and internal consistency [5]. Each of the three EMC scales has its own identified factor structure, allowing for individual use at various stages or collectively during the postpartum period [5]. However, the original version of the EMC scales lacks reported quantitative content and face validity indicators.

In Iran, similar to other world regions, numerous tools have been validated to assess different aspects of care during childbirth. The use of these scales has highlighted a significant disparity between the care provided during this period and the desired standards in the country [13–15]. There is a lack of information regarding women’s experiences during pregnancy and postpartum in the country. A study conducted in Zahedan, Iran, revealed that almost all women (97%) received prenatal care, but half of them received inadequate prenatal care (initiated after the 4th month or with less than 4 visits received) [16]. Another study carried out in six provinces of Iran indicated that in high-risk provinces (determined by maternal mortality rates), the proportion of inadequate/intermediate prenatal care (care less than 6 visits) was significantly higher than in other areas (37% vs. 21%) [17]. These studies mainly focused on the quantity of care.

To the best of our knowledge, a validated scale for assessing Iranian women’s experiences of maternity care from pregnancy through the postpartum period has not been established in Iran. If the validity of the EMC scales is confirmed in the Iranian context, their use could play a pivotal role in identifying and enhancing strengths, addressing weaknesses, and ultimately improving the quality of maternity care in our society.

## Methods

### Study aim and design

This cross-sectional methodological research aimed to determine the validity and reliability of three scales that measure maternity care experiences throughout pregnancy, labour and birth, and the early postnatal period in Iranian women.

### Participants

The participants were women aged 18 or above who had given birth one to six months prior, with seemingly healthy singleton newborns with a gestational age of 37 weeks or more. Exclusion criteria included women with any of the following characteristics: less than five years of education, mental disabilities, hearing impairments, speech impairments, recent traumatic experiences (such as divorce or loss of close relatives within the past three months), undergoing treatment with antidepressant medications, or a history of substance abuse. Women who had a previous cesarean section or an elective cesarean in their most recent delivery were also excluded because EMC-LB cannot be used for women with no experience of labour pain in their most recent delivery. In Iran, almost all women (98.4%) with a history of cesarean section have cesarean delivery in their subsequent deliveries [18].

### Sample size

For factor analysis, it is recommended to have a sample size of 10 participants per item [19]. With 12 items in each of the scales and applying the design effect related to cluster sampling of 1.5, a total of 180 samples were needed. The study included 540 participants, of whom 216 were randomly selected for exploratory factor analysis. The remaining 324 participants were used for confirmatory factor analysis and other analyses.

### Recruitment

Women for the study were selected from public health centres in Tabriz, Iran, where about 74% of recently delivered women are covered [20]. Considering the highest social and economic dispersion, 16 centres were randomly selected from densely populated health centres. The first author and principal investigator, EJ, visited the selected centres and extracted the information of all recently delivered women from the Integrated Electronic Health Records system (known as SIB). She also contacted all potentially eligible women by phone, explained the research and confidentiality of information obtained, checked eligibility criteria using a checklist, obtained verbal informed consent from eligible volunteers, and conducted interviews to complete the study questionnaires.

### Data collection tools

#### Demographic and fertility-related questionnaire

This researcher-designed questionnaire included age, gestational age at childbirth, education, occupation, income adequacy for living expenses, number of pregnancies and childbirths, intent for pregnancy, type of childbirth, place of childbirth (almost all women [> 99%] in the urban areas of the country are delivered in hospitals; private/public/social security/military hospitals), preferred type of childbirth, participation in childbirth preparation classes, and the primary source of information about childbirth.

#### Experiences of Maternity Care (EMC)

This measure assesses women’s experiences of maternity care in three stages: pregnancy, labour and birth, and the early postnatal period. Each stage consists of 12 items scored on a Likert scale ranging from 0 (strongly disagree) to 4 (strongly agree) with some items being reverse-scored. The pregnancy scale comprises five subscales: care appraisal (PR-CA), information-giving (PR-InG), communication (PR-Com), continuity (PR-Con), and antenatal checks (PR-ACh). The labour and birth scale includes two subscales: care quality (LB-CQ) and care needs (LB-CN). The postnatal scale consists of three subscales: adequacy of postnatal care (PN-APC), health professional communication (PN-HPC), and individualized care (PN-InC). Scores for each scale and subscale are calculated by summing up the relevant item scores, with higher scores indicating a more positive care experience [5].

#### Childbirth Experience Questionnaire Version-2 (CEQ-2)

This questionnaire comprises 23 items categorized into four domains: own capacity, professional support, perceived safety, and participation, with four-point Likert options (scored 1 to 4). Average scores are calculated for each domain and overall experience, with higher scores indicating a more positive childbirth experience [21].

### Procedure

The psychometric properties of the scales were determined through translation, content, face, structure, convergent and divergent validities, as well as test-retest reliability and internal consistency.

### Translation process

The translation process followed the Forward and Backward method. Initially, two proficient individuals in Persian and English who were knowledgeable in the subject matter independently translated the scales from English to Persian, taking cultural concepts into account. Then, two of the paper’s authors (SMAC, EJ), proficient in both languages, reviewed the initial translated versions and created an initial Persian version. After that, two other translators, who were not involved in the previous stage and had not seen the original version, independently translated the initial Persian version into English. The final Persian version was developed by an expert panel from the research team.

### Face validity

Face validity was assessed through both qualitative and quantitative methods. Qualitative assessment involved in-person interviews with 10 women who had recently given birth to evaluate the understanding of words and phrases, and potential misinterpretation of items. Additionally, 10 women rated item importance on a five-point Likert scale, ranging from “not important at all” (1) to “very important” (5). The impact score was calculated using the formula: Impact score = Frequency (%) × Importance. A score above 1.5 was considered acceptable for the validity [22].

### Content validity

Content validity was assessed qualitatively and quantitatively. In the qualitative evaluation, experts assessed clarity, simplicity, grammar, word choice, item relevance, item placement, and scale completion time. The quantitative evaluation was based on the opinions of 16 experts and the calculation of two indices: the Content Validity Index (CVI) and the Content Validity Ratio (CVR). In determining CVR, the experts evaluated each item on a four-point Likert scale (from essential to not useful). According to the Lawshe table, a CVR greater than 0.49 confirms the necessity of each item. For assessing CVI, three criteria—clarity, simplicity, and relevance of the items—were evaluated on a four-point Likert scale, and a CVI above 0.79 was considered valid [23].

### Construct validity

Construct validity indicates whether the items of a scale are reasonably grouped together to measure the intended purpose [19]. This validity was assessed using EFA and CFA.

To conduct EFA, the correlation matrix between items within each scale was computed, and factor extraction was performed using principal axis factoring. Subsequently, factor rotation and Quatrimax oblique rotation were employed. The consistency of these factors with the concept and dimensions of maternal care experiences was then evaluated. Model adequacy was assessed using the Kaiser-Meyer-Olkin (KMO) measure and Bartlett’s Test. Eigenvalue and scree plot methods were used to determine the number of factors, with a factor loading cut-off of 0.30 considered [24].

CFA was employed to evaluate the structure of the factors identified through EFA. Model fit was evaluated based on the following indicators: Root Mean Square Error of Approximation (RMSEA) ≤ 0.08, Standardized Root Mean Square Residual (SRMR) ≤ 0.08, Normed Chi-Square (χ2/df) < 5, Comparative Fit Index (CFI ≥ 0.90), Normed Fit Index (NFI) ≥ 90, Goodness-of-Fit (GFI) ≥ 90, and Non-Normed Fit Index (NNFI) ≥ 0.90 [25].

The correlation of all EMC scales and subscales with each other was assessed using the Pearson correlation test, with correlations interpreted as weak (*r* = 0.1–0.2), fair (*r* = 0.3–0.5), moderate (*r* = 0.6–0.7), or very strong (*r* = 0.8 and above) [26].

### Convergent validity

Convergent validity refers to the degree of correlation between two tests that assess closely related constructs [27]. The CEQ-2 was used to assess the convergent validity of EMC-LB, while an appropriate scale was not found for the other two EMC scales.

### Divergent validity

Divergent validity was examined by calculating the Pearson correlation coefficient between the scores of EMC scales and subscales with women’s age. It was anticipated that there would be no significant relationship between scores of EMC scales and the women’s age [5].

### Reliability

Test-retest reliability was assessed by having 15 randomly selected women complete the scales twice, two weeks apart, and the intraclass correlation coefficient (95% confidence interval [CI]) was calculated. Additionally, Cronbach’s alpha was utilized to determine internal consistency. An intraclass correlation coefficient above 0.80 and a value of Cronbach’s Coefficient alpha greater than 0.70 were considered indicative of suitability [24].

### Statistical analysis

Statistical analysis was conducted using IBM SPSS version 25 (IBM Corp, Armonk, NY, USA) and STATA version 18 (Stata Corp, College Station, Texas, USA). Participant characteristics were presented as numbers (percentage) for qualitative variables and as mean (standard deviation) for normally distributed continuous variables.

### Ethical considerations

Ethical approval was obtained from the Ethics Committee of Tabriz University of Medical Sciences, Tabriz, Iran (IR.TBZMED.REC.1401.285). Permission to use the EMC scales was obtained via email from Professor Maggie Redshaw, the first and corresponding author of the original scale development article affiliated with the University of Oxford, UK. Verbal informed consent was obtained from all participants prior to their enrolment in the study. Participants were assured of the confidentiality of their information and their right to withdraw from the study at any time.

## Results

### Descriptive results

Recruitment was carried out from December 2022 to June 2023. The mean age of the participants was 28.7 years (SD 6.1), with 31.5% being primiparous. Further participant characteristics are presented in Table 1.

**Table 1:** Characteristics of the study participants (*n* = 540)

Characteristics	*n* (%)
Age (years)	28.7 (6.1)^a^
Gestational age at birth (weeks)	38.7 (1.0)^a^
Education	
Elementary or secondary school	200 (37.0)
High school or diploma	250 (46.3)
College	90 (16.7)
Paid employment	30 (5.6)
Income	
Not enough at all	160 (29.6)
Relatively enough	322 (59.6)
Quite enough	58 (10.7)
Primigravida	154 (28.5)
Primiparous	170 (31.5)
Planning for pregnancy	
Planned	378 (70)
Mistimed	27 (5)
Unwanted	135 (25)
Hospitalizations during the most recent pregnancy (yes)	38 (7)
Cause of hospitalization	
Preterm labour	18 (47.4)
COVID-19	8 (21.1)
Hypertension	7 (18.4)
Other reasons	5 (13.2)
Type of delivery	
Non-instrumental vaginal delivery	534 (98.9)
Emergency cesarean section	6 (1.1)
Place of birth	
Private hospital	137 (25.4)
Public hospital	148 (27.4)
Social Security Hospital	93 (17.2)
Military Hospital	162 (20.0)
Preferred to have a vaginal delivery	404 (74.8)
Participated in birth preparation classes	82 (15.2)
The main source of information about childbirth	
Midwife	121 (22.7)
Obstetrician	72 (13.5)
Family, friends, and media	34 (6.4)
Previous birth	306 (57.4)

^a^Mean (SD)

The mean, standard deviation, skewness, and kurtosis of the scale scores and their subscales are outlined in Table 2. In all instances, the skewness was less than 2.5, and the kurtosis was less than 4, indicating a normal distribution.

**Table 2:** Characteristics of each of the experience of maternity care (EMC) scales and subscales (*n* = 216^a^)

Scale and subscale scores	Mean (SD)	Obtained score range	Skew	Kurtosis
**Pregnancy scale (0–48)**	35.7 (10.1)	6–48	-0.75	-0.03
Antenatal checks (0–8)	6.4 (1.7)	1–8	-0.90	-0.15
Care appraisal (0–12)	9.0 (3.0)	0–12	-0.95	0.31
Information-giving (0–8)	5.1 (2.6)	0–8	-0.58	-0.88
Communication (0–12)	8.1 (3.3)	0–12	-0.52	-0.70
Continuity (0–8)	7.0 (1.7)	1–8	-2.02	3.65
**Labour & birth scale (0–48)**	31.3 (12.2)	2–48	-0.72	-0.46
Care quality (0–28)	20.4 (6.8)	2–28	-1.17	0.45
Care needs (0–20)	10.9 (6.3)	0–20	-0.16	-1.18
**Postnatal scale (0–48)**	36.1 (10.7)	0–48	-1.02	0.66
Adequacy of postnatal care (0–16)	12.6 (4.2)	0–16	-1.29	0.79
Health professionals Communication (0–16)	11.3 (4.3)	0–16	-0.78	-0.21
Individualised care (0–16)	12.1 (3.6)	0–16	-1.18	1.32

^a^In split-half exploratory factor analysis dataset

A higher score indicates a better experience of maternity care

### Face and content validity

During face validity assessment, all items were found to be proportionate, unambiguous, and easy to understand, with impact scores varying from 3.5 to 4.9. In the content validity assessment, all items had a CVI and CVR above 0.8 (Supplementary Table S1), indicating that none needed to be excluded.

### Construct validity

In the EFA, all items in the three scales were loaded with the same factors as the scales were initially developed. The Kaiser-Meyer-Olkin measures for EMC-PR, EMC-LB, and EMC-PN were 0.88, 0.93, and 0.88, respectively. Bartlett’s test was statistically significant (*p* < 0.001). According to the eigenvalues, EMC-PR accounted for 66% of the variance with a five-factor structure (Table 3), EMC-LB explained 57% of the variance with a two-factor structure (Table 4), and EMC-PN explained 62% of the variance with a three-factor structure (Table 5).

**Table 3:** Factor structure of the experiences of maternity care (EMC) during pregnancy (*n* = 216)

Items	Factor 1	Factor 2	Factor 3	Factor 4	Factor 5
Factor 1: Care appraisal (CA)					
CA1. Overall, I was very pleased with the care I received in pregnancy	0.971				
CA2. During pregnancy, I did not feel well cared for by health professionals^a^	0.665				
CA3. My care provider(s) gave me all the information I needed	0.595				
Factor 2: Information-giving (InG)					
InG1. I was not given enough explanations about antenatal scans and tests^a^		0.821			
InG2. I was not given enough information to make decisions about my antenatal care^a^		0.821			
Factor 3: Communication (Com)					
Com1. Antenatal appointments were too short to discuss any concerns about my pregnancy^a^			0.837		
Com2. I was not involved enough in decisions about my antenatal care^a^			0.728		
Com3. Health professionals did not always talk to me in a way I could understand^a^			0.494		
Factor 4: Continuity (Con)					
Con1. I was happy with the number of health professionals who cared for me during my pregnancy				0.807	
Con2. I always saw the same midwife/doctor for my antenatal checks				0.807	
Factor 5: Antenatal checks (Ach)					
ACh1. I felt I had the right number of antenatal checks with the midwife/doctor					0.41
ACh2. I would have liked more antenatal checks and scans^a^					0.41
TVE (%Variance explained)	45.3%	9.2%	5.8%	3.8%	1.9%
Cumulative TVE	45.3%	54.5%	60.3%	64.2%	66.0%
Kaiser-Meyer-Olkin (KMO)	0.880				
Bartlett’s Test	< 0.001				
McDonald omega 0.90 (Total)	0.82	0.81	0.72	0.23	0.61

^a^Items are reverse-scored

**Table 4:** Factor structure of the experiences of maternity care (EMC) during labour and birth (*n* = 216)

Items			Factor 1	Factor 2
Factor 1: Care quality (CQ)				
CQ1. I had confidence and trust in the staff caring for me			0.913	
CQ2. Staff communicated well with me during labour and birth			0.855	
CQ3. I had the best possible care during labour and birth			0.811	
CQ4. I felt safe in the labour and birth environment			0.774	
CQ5. I was treated as an individual by staff			0.709	
CQ6. Everything was explained to me well during labour and birth			0.573	
CQ7. I did not mind being looked after by midwives or doctors I had not met before			0.256	
Factor 2: Care needs (CN)				
CN1. I needed more staff support during labour and birth^a^				0.554
CN2. The staff could have done more to help me to feel in control of my labour and birth^a^				0.917
CN3. I was not involved enough in decisions about procedures that were carried out (e.g. breaking waters, epidural, caesarean section)^a^				0.812
CN4. Health professionals left me alone more than I would have liked^a^				0.520
CN5. I felt that my pain relief needs were not managed well^a^				0.493
TVE (%Variance explained)			0.511	0.056
Cumulative TVE			0.511	0.568
Kaiser-Meyer-Olkin (KMO)	0.927			
Bartlett’s Test	< 0.001			
McDonald omega	0.92 (Total)		0.89	0.86

^a^Items are reverse-scored

**Table 5:** Factor structure of the Experiences of Maternity Care (EMC) during postnatal (*n* = 216)

Items			Factor 1	Factor 2	Factor 3
Factor 1: Adequacy of postnatal care (APC)					
APC1. I received enough care and attention from staff on the postnatal ward			0.887		
APC2. I was treated as an individual by midwives/doctors after the birth			0.879		
APC3. After I had given birth, health professionals treated me as though I was no longer important^a^			0.750		
APC4. I stayed in hospital as long as I wanted after the birth			0.578		
Factor 2: Health professional communication (HPC)					
HPC1. I was not given the advice and information I needed by health professionals after my baby was born^a^				0.904	
HPC2. I had enough information from health professionals about how to care for my baby				0.766	
HPC3. There was not enough time to talk over my concerns with health professional^a^				0.726	
HPC4. I was able to build a good relationship with the healthcare professionals I saw after coming home				0.566	
Factor 3: Individualised care (InC)					
InC1. Overall, I was very pleased with the quality of my postnatal care					0.904
InC2. The postnatal care I received did not meet the needs of me and my baby^a^					0.895
InC3. I had all the checks I needed after the birth					0.512
InC4. After the birth of my baby, I knew who to contact if I had questions or concerns					0.433
TVE (%Variance explained)			49.4%	7.7%	5.3%
Cumulative TVE			49.4%	57.1%	62.3%
Kaiser-Meyer-Olkin (KMO)	0.880				
Bartlett’s Test	< 0.001				
McDonald omega	0.91 (Total)		0.87	0.86	0.81

^a^Items are reverse-scored

CFA was performed on a second dataset (*n* = 324) for the three EMC scales identified in EFA, each containing 12 items. All three scales had acceptable indices of RMSEA ≤ 0.08, SRMR ≤ 0.08, NFI ≥ 0.90, NNFI ≥ 0.90, CFI ≥ 0.90, and GFI ≥ 0.90 (Table 6). The model fit estimates indicated acceptable fit across various indices, confirming their factorial structure.

**Table 6:** Confirmatory factor analysis model fit statistics for each of the experiences of maternity care (EMC) scales (*n* = 324)

Model fit statistics	Pregnancy	Labour and birth	Postnatal
Factor N	5	2	3
χ2/df	171.9/38 (4.52)	284.78/53 (5.37)	206.80/51 (4.05)
RMSEA (95% CI)	0.07 (0.06–0.90)	0.08 (0.06–0.09)	0.08 (0.04–0.09)
SRMR	0.07	0.05	0.07
NFI	0.91	0.90	0.94
NNFI	0.90	0.90	0.91
CFI	0.93	0.92	0.95
GFI	0.99	0.95	0.94

*χ2/df *Ratio of the chi-square to the degrees of freedom, *RMSEA *Root means square error of approximation, *SRMR *Standardized Root Mean Square Residual, *NFI *Normed Fit Index, *NNFI *Non-normed Fit Index, *CFI *Comparative Fit Index, *GFI *Goodness of Fit Index

**Fig. 1:**
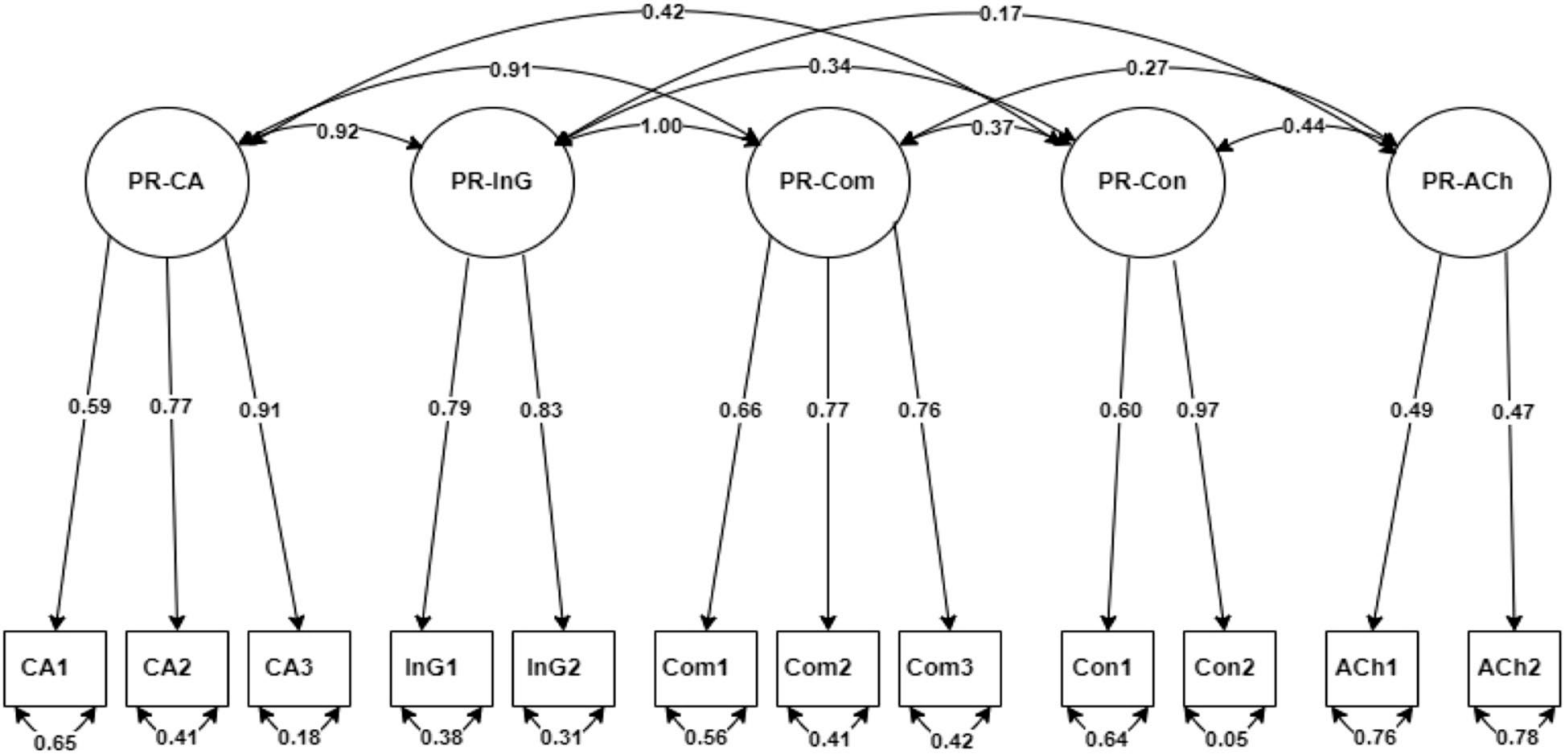
CFA factor loading for the pregnancy scale.  PR: Pregnancy, CA: Care appraisal, InG: Information-giving, Com: Communication, Con: Continuity, Ach: Antenatal checks

**Fig. 2:**
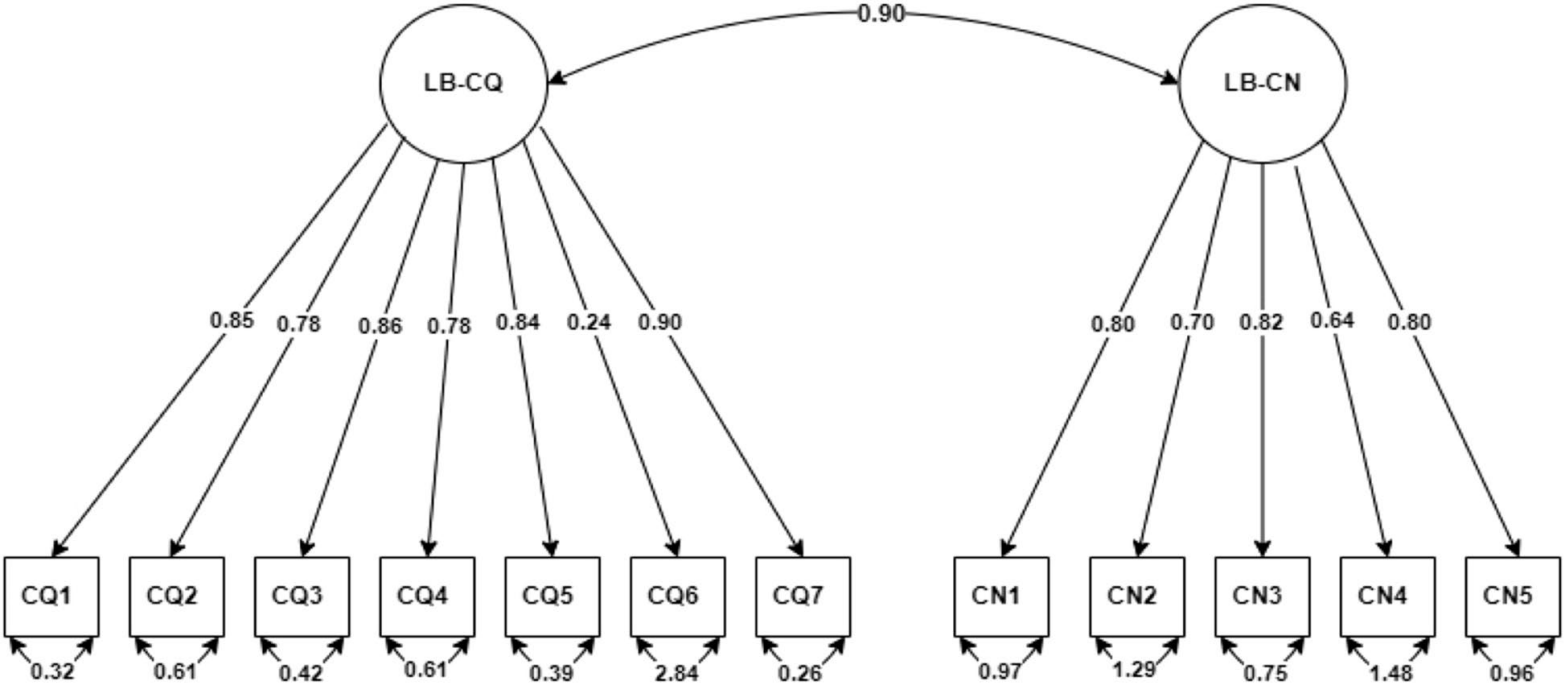
CFA factor loading for labour and birth scale LB: Labour and birth, CQ: Care quality, CN: Care needs

**Fig. 3:**
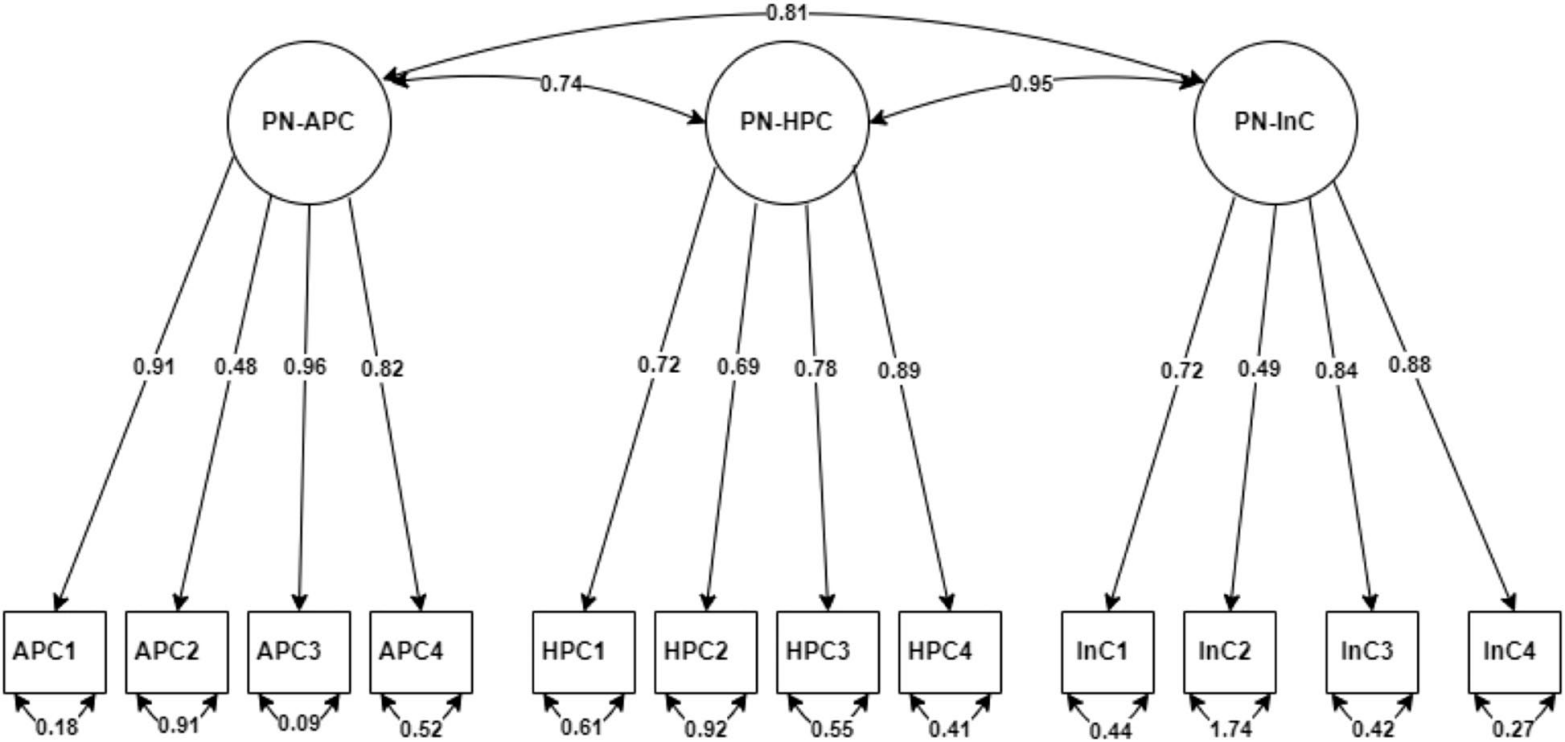
CFA factor loading for labour and birth scale.  PN: Postnatal, APC: Adequacy of postnatal care, HPC: Health professional communication, InC: Individualised care

A path diagram with standardized coefficients was created for the pregnancy scale (Fig. 1), labour & birth scale (Fig. 2), and postnatal scale (Fig. 3).

The abbreviated names correspond to the names presented in Table 3.

The arrows leading from the factor (subscale) in the circle to each item in the box represent the coefficient weight of the factor on the individual item. The value with double-headed arrows below each item shows the variance estimate of the factor. The double-headed arrows between the factors show the covariance between factors.

The abbreviated names correspond to the names presented in Table 4.

The abbreviated names correspond to the names presented in Table 5.

All EMC scales showed strong or very strong correlations with their respective subscales, with Pearson’s r values ranging from 0.72 to 0.96. However, the EMC-pregnancy scale displayed a weaker correlation of 0.56 with the continuity sub-scale. The EMC-pregnancy exhibited weak to fair correlations (ranging from 0.28 to 0.38) with the EMC labour & birth and EMC postnatal scales, as well as their respective subscales. EMC labour & birth and EMC postnatal scales showed poor or fair correlations with four subscales of EMC pregnancy (ranging from 0.29 to 0.38) and no significant correlation with the subscale of continuity. Moderate correlations (ranging from 0.57 to 0.65) were found between the EMC labour & birth and EMC postnatal scales, as well as between each scale and the subscales of the other scale (Supplementary Table S2).

### Convergent validity

In terms of convergent validity, Pearson’s test showed a significant correlation (*r* = 0.82 [95% CI 0.78–0.86], *P* < 0.001) between EMC-LB and CEQ-2. Significant correlations were also found between all subscales of EMC-LB and CEQ-2, with correlation coefficients ranging from 0.60 to 0.80 (*P* < 0.001). The strongest correlation was observed between the subscales of EMC-LB-care quality and CEQ-Professional support (*r* = 0.80), while the weakest correlation was found between the subscale of EMC-LB-care quality and CEQ-own capacity (*r* = 0.60) (Table 7).

**Table 7:** Pearson’s r correlations between EMC labour & birth scale and subscales and CEQ-2 scale and subscales (*n* = 324)

CEQ-2 scale & subscales	EMC-LB	EMC-LB care quality	EMC-LB care needs
CEQ-2	0.82	0.78	0.79
Own capacity	0.64	0.60	0.63
Professional support	0.82	0.80	0.78
Perceived safety	0.75	0.73	0.70
Participation	0.79	0.74	0.77

*EMC *Experiences of maternity care, *EMC-LB *EMC labour & birth, *CEQ-2 *Childbirth Experience Questionnaire version 2, All *P* values are less than 0.001

### Divergent validity

Pearson’s test did not indicate a significant correlation (Ps > 0.05) between women’s age and the scores of EMC scales and their subscales (Supplementary Table S3).

### Reliability

The Cronbach’s alpha coefficients for EMC-PR, EMC-LB, and EMC-PN were 0.91, 0.92, and 0.92, respectively. The coefficients for all subscales of the scales were above 0.70, except for Antenatal checks in EMC-PR, which was 0.33. The two items of the Antenatal checks subscale showed a significant correlation, but a weak correlation (*r* = 0.23). In the test-retest, the intraclass correlation coefficient (95% CI) for EMC-PR was 0.88 (0.64–0.96), for EMC-LB was 0.93 (0.78–0.98), and for EMC-PN was 0.95 (0.88–0.98) (Supplementary Table S4).

## Discussion

The results of the current study demonstrated the EMC scales as robust, valid, and reliable tools for assessing the maternity care experiences of Iranian women. The EMC scales used in this study demonstrated desirable content and face validity. CFA confirmed the structures derived from EFA, and the convergent validity of EMC-LB with CEQ-2 was established. Additionally, the study confirmed the divergent validity of the EMC scales by showing no relationship with women’s age. Furthermore, the scales were found to be reliable regarding internal consistency and test-retest reliability.

In our study, the KMO value of the EMC scales was satisfactory, and the extracted structures accounted for 57–66% of the cumulative variance. In the original version of EMC, similar scale structures had been extracted and confirmed, but the KMO value and explained variance were not reported [5]. In a study conducted in Norway, eight translated versions of EMC-LB were used, but no information was provided on the psychometric indices [28]. KMO and explained variance were not reported for the Pregnancy and Maternity Care Patients’ Experiences Questionnaire [9] and Clark et al.‘s scales [10], which have a similar purpose. The other measure, ESEM [11], consists of 30 items in three scales of pregnancy, intrapartum, and postnatal, with structures explaining 55%, 62%, and 70% of the cumulative variance, respectively. In comparison to our study, these percentages of explained variances are lower for pregnancy, almost equal for intrapartum, and slightly higher for postnatal. It appears that the five-factor structure of EMC-PR encompasses more diverse aspects than the one-factor structure of ESEM-pregnancy. For instance, ESEM-pregnancy lacks items related to sonography and continuity of care. In ESEM-intrapartum and EMC-LB, there are aspects of care that do not exist in the other, such as the absence of any items about pain relief and continuity of care in ESEM-intrapartum and the absence of physical aspects of care in EMC-LB. ESEM-postnatal includes items about participation in decision-making, education on common problems during the postnatal period, and physical aspects of care, but it has no item about the first weeks of care at home. The Pregnancy and Childbirth Questionnaire (PCQ) has a two-factor structure for pregnancy and a one-factor structure for childbirth, explaining 53% and 56% of the cumulative variance, respectively. However, this measure does not address the quality of postnatal care [12].

Despite the subscale loading variance for the *Continuity* and *Antenatal checks* subscales in the EMC-PR scale falling below the recommended threshold of 5%, we decided to retain these subscales for several reasons: Firstly, keeping these subscales enabled us to maintain alignment with the original scale, preventing the removal of associated items. Secondly, these subscales play a critical role in capturing specific aspects of the construct under investigation. Their importance is underscored by evidence from Cochrane reviews, emphasizing the pivotal role of midwifery-led continuity of care [29] and standard antenatal check-ups [30] in enhancing maternal and neonatal outcomes. Additionally, the World Health Organization (WHO) advocates for both continuity of care and a minimum of eight antenatal contacts, incorporating effective interventions and tests, as essential components of antenatal care to promote a positive pregnancy experience [31].

In the EMC-LB subscale, the item of *“I did not mind being looked after by midwives or doctors I had not met before”* displayed a factor loading of 0.256, falling below the conventional threshold of 0.300. Nevertheless, we chose to keep this item in our analysis for two main reasons. Firstly, to maintain consistency with the original questionnaire, and secondly, due to the paramount importance of continuity of care in maternal and newborn health. A Cochrane systematic review highlighted the significant advantages associated with midwifery-led continuity of care, leading to enhanced woman satisfaction and improved outcomes for both women and newborns compared to other care models [32]. Additionally, based on the evidence, the WHO recommends the implementation of this care model in settings with well-functioning midwifery programmes [33]. However, only New Zealand has fully embraced it as a standard national practice, as documented in the literature [34]. Therefore, the inclusion of such an item in the assessment of care quality holds particular relevance, especially within the societal context of Iran. Although Iran places a strong emphasis on enhancing childbirth satisfaction as part of its new pronatalist population policy [35, 36], the adoption of this care model remains limited [29, 37, 38].

Our study’s average scores of EMC-PR and EMC-PN are almost identical to those reported in a study conducted in England to develop the original version [5]. However, our study reveals a lower score for EMC-LB, likely due to the absence of a woman-centred approach in childbirth services in Iran [39–42]. A study within our research context recognized the necessity for effective interventions in childbirth care, including the presence of a companion, respectful care, effective communication, education, responsiveness to needs, participation in decision-making, reduction of unnecessary interventions, and provision of pain relief options [43].

The strong correlation observed between EMC-LB and CEQ-2, a validated scale for assessing childbirth experience [21], reinforces the validity of EMC-LB. This finding is consistent with the original EMC study, which also found a significant correlation between this measure and a question about the right to choose in maternity care. Measures assessing similar constructs are anticipated to exhibit a strong correlation [44]. EMC-LB and CEQ share similar concepts, including pain relief, personal control, safety, confidence, information, communication, shared decision-making, and support. Notably, the variance explained by the EMC-LB scale with 12 items surpasses that of CEQ-2 with 23 items (57% versus 43%) [21]. This could be attributed to EMC-LB’s emphasis on crucial concepts such as continuity of care [45] and individualized care [8], in addition to the shared concepts. The strong positive correlation between the EMC-LB subscale in care quality and CEQ professional support indicates the importance of receiving support from professional care providers in women’s experience of labour and birth care quality, as highlighted in other studies. A systematic review emphasizes the importance of having competent, reassuring, kind, and supportive clinical staff to facilitate a positive childbirth experience [46].

In our study, Cronbach’s alpha for all EMC scales and subscales, except for the Antenatal checks’ subscale in EMC-PR, exceeded the minimum acceptable threshold. However, in the original version, four subscales related to EMC-PR, including Antenatal checks, had Cronbach’s alpha values below the acceptable threshold [5]. The researchers did not provide an opinion on this matter. The lower internal consistency of the Antenatal checks’ subscale may be due to its limited number of items (only two). It is important to note that when assessing the internal consistency of a two-item scale, Pearson’s r is preferred over Cronbach’s alpha, with an r value greater than 0.15 considered satisfactory [47]. In this instance, Pearson’s r value was 0.23. Additionally, the dual nature of the “I would have liked more antenatal checks and scans” item may contribute to its inconsistency with the “I felt I had the right number of antenatal checks with the midwife/doctor” item. A recent systematic review has highlighted a shift in the culture of prenatal care management in many low- and middle-income countries, where ultrasound technology has replaced important components of clinical exams [48]. In Iran, the indiscriminate use of medical technology, especially in urban areas, is widespread. For instance, a study in the city of Urmia-Iran revealed an average of 5.9 ultrasound scans per woman during pregnancy, with most women overestimating the diagnostic power of ultrasound and expressing only a few negative feelings about it [49].

In the present study, all EMC scales and their subscales had high test-retest reliability, except for the “EMC-PR-information giving” subscale, which had an ICC of 0.69. It is possible that receiving prenatal care from multiple caregivers may have interfered with responses to the items in this subscale. Some assessments of the reliability of maternity care experience scales [5, 10, 12] have relied only on Cronbach’s alpha, while according to the COnsensus-based Standards for the selection of the health status Measurement INstruments (COSMIN), assessing ICC is of high importance [50]. Test-retest reliability has not been mentioned for the original scale [5]. Moreover, half of the 16 Pregnancy and Maternity Care Patients’ Experiences Questionnaire scales also had ICC values less than 0.8, which could be due to the response burden resulting from the large number of items (145 items) in these scales [9].

The validity indicators, especially content and face validity, have been inadequately dealt with in psychometric evaluations of maternity care experience scales, as indicated by two recent systematic reviews [7, 8]. It is argued that when claiming that a scale is designed for measuring women-centred maternity care experiences, it should incorporate women’s participation in decision-making for relevant, understandable, and comprehensive items [8]. Content validity is the most important characteristic of a patient-reported experience measure. It ensures that the scale’s content precisely reflects the phenomenon that the scale’s user intends to measure [51]. In this study, we attempted to investigate more psychometric indicators and present a more comprehensive report of the relevant results.

Maternity care managers and providers, especially midwives, are expected to use these validated short scales to assess the quality of current care. Furthermore, researchers are encouraged to employ these tools to explore the effects of different care models in various studies.

### Strengths and limitations

This study holds significant importance for several reasons. Firstly, it assessed the EMC scales’ psychometric properties for the first time in a cultural context outside of England. We reported comprehensive validity and reliability indices in this paper. Also, we executed exploratory and confirmatory factor analyses on two distinct random samples of the participants. The study was conducted among randomly selected women covered by the health centers of Tabriz, the capital city of the fifth-largest province in Iran. Given that the centers cover the majority of postpartum women, the results might be applicable to all eligible women in the city. Most of the fertility indicators in this city closely align with the mean of the indicators in urban areas of the country. Therefore, the results may be generalizable to all urban areas of the country.

This study’s results may not apply to women who have had multiple pregnancies, premature births, or those who have infants with disabilities, as these groups were not included in the study. Furthermore, due to the small number of participants with pregnancy complications, a history of illness, or those who underwent emergency cesarean sections, the results may not be generalizable to these groups and they may have different experiences.

## Conclusions

The Persian version of all three EMC scales have demonstrated robust psychometric properties for assessing the experiences of maternity care among urban Iranian women. These scales are valuable for assessing current care quality and investigating the effects of different care models in various research studies. Therefore, these scales can aid in advancing maternal health programs and policies. However, future studies should examine how applicable these findings are to rural women, women with high-risk pregnancies and deliveries, as well as women with infants at risk.

### Supplementary Information

Supplementary Material 1.

Supplementary Material 2.

Supplementary Material 3.

## Data Availability

All de-identified participant datasets will be available for research purposes to researchers affiliated with academic institutions and others upon reasonable request from the corresponding author immediately after the results are published.

## Abbreviations

CFA: Confirmatory factor analysis
CEQ-2: Childbirth Experience Questionnaire Version-2
CVI: Content validity index
CVR: Content validity ratio
EFA: Exploratory factor analysis
EMC: Women’s Experiences of Maternity Care
ESEM: Women’s Experience of Maternity Care
LB: Labour & Birth Scale
PN: Postnatal Scale
PR: Pregnancy Scale
