# Supplementary material for: Psychometric properties of the experiences of maternity care scale among Iranian women
Source: BMC Health Serv Res. 2024 May 11;24:619. doi: 10.1186/s12913-024-11065-1 (PMC11088168; doi:10.1186/s12913-024-11065-1)
Supplement: Supplementary file 1 — Supplementary Material 1. [file 12913_2024_11065_MOESM1_ESM.docx]

**Demographic and fertility-related questionnaire**

| Participant code: ……………………………... |
| --- |
| Date of completion of the questionnaire: ……………………. |
| Covered by health center No: ………………………………... |
| Mobile number: .................................. |
| Mobile number of husbands, mother, or sister: …………. |

| Demographic characteristics | | | | | |
| --- | --- | --- | --- | --- | --- |
| Year of birth: ........................... | | |  |  |  |
| Education level: | Elementary school □ | | Secondary school□ | High school □ | Diploma □ |
|  | Collage □ | |  |  |  |
| Job: | Housekeeper □ | | Employment outside the home □ | Employment inside the home □ |  |
| Income: | Not enough at all □ | | Relatively enough □ | Quite enough □ |  |
| Fertility-related characteristics | | | | | |
| Gravida: …… | | Parity: …… | The number of abortions: ….. | The number of living children: ….. | The number of dead children: …. |
| Data about recent pregnancy & Birth  The date of the first ultrasound in pregnancy: ……………………………….  Gestational age at the first sonography: ……. | | | | | |
| Gestational age at delivery: ……………………………….. | | | | | |
| Date of delivery: ……………………………………….. | | | | | |
| Gravida: …… | | Parity: …… | The number of abortions: ….. | The number of living children: ….. | The number of dead children: …. |
| Planning for pregnancy: | | Planned □ | Mistimed□ | Unwanted □ |  |
| History of infertility: | | Yes/primary infertility□ | Yes/secondary infertility□ | No□ |  |
| Type of delivery: | | Normal vaginal delivery □ | Emergency cesarean section□ |  |  |
| Place of delivery: | | Private hospital □ | Public hospital □ | Social Security Hospital □ | Military Hospital □ |
| Preferred mode of delivery in pregnancy | | Normal vaginal delivery □ | cesarean section □ |  |  |
| Participation in childbirth preparation classes | | Yes □ | No □ |  |  |
| Main source of information about childbirth | | Midwife □ | Obstetrician □ | Family, friends and media □ | Previous birth □ |
