# Supplementary material for: Psychometric properties of the experiences of maternity care scale among Iranian women
Source: BMC Health Serv Res. 2024 May 11;24:619. doi: 10.1186/s12913-024-11065-1 (PMC11088168; doi:10.1186/s12913-024-11065-1)
Supplement: Supplementary file 2 — Supplementary Material 2. [file 12913_2024_11065_MOESM2_ESM.docx]

|  | **When I am thinking of my prenatal care** | **Strongly agree** | **Agree to some extent** | **Neither agree nor disagree** | **Disagree to some extent** | **Strongly disagree** |
| --- | --- | --- | --- | --- | --- | --- |
| 1 | The numbers of prenatal cares I received from my midwife/ physician was adequate |  |  |  |  |  |
| 2 | The care provider (s) gave me all the information I needed |  |  |  |  |  |
| 3 | A midwife/physician always took care of me regularly during my pregnancy |  |  |  |  |  |
| 4 | Care providers sometimes talked to me in a way that I did not understand them |  |  |  |  |  |
| 5 | The time allotted for my prenatal care sessions was so short that I did not have the opportunity to discuss all of my pregnancy worries and concerns |  |  |  |  |  |
| 6 | I was not involved enough in making decisions about my own prenatal care |  |  |  |  |  |
| 7 | I was satisfied with the number of self-care providers during my pregnancy |  |  |  |  |  |
| 8 | Enough information about ultrasounds and pregnancy tests was not given to me |  |  |  |  |  |
| 9 | Enough information to decide on pregnancy care was not given to me |  |  |  |  |  |
| 10 | I wish I had more pregnancy tests and ultrasounds |  |  |  |  |  |
| 11 | During my pregnancy, I did not feel that I was well cared for by providers |  |  |  |  |  |
| 12 | In general, I was very satisfied with the care I received during my pregnancy |  |  |  |  |  |
|  | **When I am thinking of cares provided during labor pains and my delivery** | **Strongly agree** | **Agree to some extent** | **Neither agree nor disagree** | **Disagree to some extent** | **Strongly disagree** |
| 1 | The staff had a good relationship with me during labor pains and delivery |  |  |  |  |  |
| 2 | During labor pains and delivery, I needed more staff support than they provided |  |  |  |  |  |
| 3 | During labor pains and delivery, everything was well explained to me |  |  |  |  |  |
| 4 | The staff treated me according to my individual needs |  |  |  |  |  |
| 5 | I was not involved enough in the decisions related to the delivery process (such as rupture of the amniotic sac, performing cesarean section, etc.) |  |  |  |  |  |
| 6 | The health professionals left me alone more than as I wanted |  |  |  |  |  |
| 7 | I felt that my need for pain relief was not well met |  |  |  |  |  |
| 8 | I felt safe in the delivery environment |  |  |  |  |  |
| 9 | The staff could have helped me more than they did to control my labor pains and delivery |  |  |  |  |  |
| 10 | I had confidence in the staff who cared for me |  |  |  |  |  |
| 11 | I did not feel bad about being cared by midwives or physicians I had never met before |  |  |  |  |  |
| 12 | I received the best possible care during labor pains and delivery |  |  |  |  |  |

|  | **When I am thinking of my prenatal care** | **Strongly agree** | **Agree to some extent** | **Neither agree nor disagree** | **Disagree to some extent** | **Strongly disagree** |
| --- | --- | --- | --- | --- | --- | --- |
| 1 | In the postpartum ward, I received adequate care and attention from the staff |  |  |  |  |  |
| 2 | After the delivery, I was hospitalized for as long as I wanted |  |  |  |  |  |
| 3 | The midwives/physicians' treatment and behavior with me after delivery was appropriate to my individual needs |  |  |  |  |  |
| 4 | After delivery, the health professionals treated me as if I was no longer important |  |  |  |  |  |
| 5 | I received enough information about how to take care of my infant from health professionals |  |  |  |  |  |
| 6 | After being discharged from the hospital, I was able to easily communicate with health professionals (to meet my needs) |  |  |  |  |  |
| 7 | After the birth of my infant, I did not receive the advice and information I needed from health professionals |  |  |  |  |  |
| 8 | There was not enough time to talk to health professionals to address my worries and concerns |  |  |  |  |  |
| 9 | All the necessary tests and examinations were done for me after delivery |  |  |  |  |  |
| 10 | After the birth of my infant, I knew who to call if I had any questions or concerns |  |  |  |  |  |
| 11 | The postpartum care I received did not meet the needs of me and my infant |  |  |  |  |  |
| 12 | In general, I was very satisfied with the quality of my postpartum cares |  |  |  |  |  |
