# Supplementary material for: Psychometric properties of the experiences of maternity care scale among Iranian women
Source: BMC Health Serv Res. 2024 May 11;24:619. doi: 10.1186/s12913-024-11065-1 (PMC11088168; doi:10.1186/s12913-024-11065-1)
Supplement: Supplementary file 3 — Supplementary Material 3. [file 12913_2024_11065_MOESM3_ESM.docx]

**Supplementary Table S1** The impact score, CVI, and CVR for items

| Pregnancy scale | | | |  | Labour and birth scale | | | | |  | Postnatal scale | | | |
| --- | --- | --- | --- | --- | --- | --- | --- | --- | --- | --- | --- | --- | --- | --- |
| Items | Impact score | CVI | CVR |  | Items | Impact score | CVI | CVR |  | | Items | Impact score | CVI | RCV |
| 1. CA3 | 4.3 | 0.98 | 1 |  | 1. CQ5 | 4.9 | 1 | 1 | |  | 1. APC1 | 4.6 | 1 | 1 |
| 2. CA2 | 4.8 | 1 | 1 |  | 2. CQ1 | 4.7 | 0.94 | 1 | |  | 2. APC3 | 4.9 | 0.94 | 0.87 |
| 3. CA1 | 4.4 | 1 | 1 |  | 3. CQ7 | 4.8 | 1 | 1 | |  | 3. APC4 | 4.7 | 0.89 | 1 |
| 4. InG1 | 4.1 | 0.83 | 0.81 |  | 4.CQ4 | 4.7 | 0.81 | 1 | |  | 4. APC2 | 3.7 | 1 | 1 |
| 5. InG2 | 4.8 | 0.94 | 0.94 |  | 5. CQ3 | 3.8 | 1 | 1 | |  | 5. HPC3 | 4.7 | 1 | 1 |
| 6. Com2 | 4 | 1 | 0.94 |  | 6. CQ2 | 4.3 | 1 | 1 | |  | 6. HPC1 | 4.2 | 0.96 | 1 |
| 7. Com3 | 4.6 | 0.87 | 0.87 |  | 7.CQ6 | 4.6 | 0.96 | 1 | |  | 7.HPC4 | 4.7 | 0.98 | 1 |
| 8. Com1 | 4.3 | 1 | 1 |  | 8. CN1 | 4.9 | 1 | 1 | |  | 8. HPC2 | 4.6 | 1 | 1 |
| 9. Con2 | 4.8 | 0.96 | 0.94 |  | 9. CN5 | 4.4 | 0.89 | 1 | |  | 9. InC4 | 4.9 | 1 | 1 |
| 10. Con 1 | 3.5 | 0.94 | 0.94 |  | 10. CN2 | 4.9 | 1 | 1 | |  | 10. InC3 | 4.7 | 1 | 1 |
| 11. Ach1 | 4 | 0.94 | 0.94 |  | 11. CN3 | 4.5 | 0.94 | 0.87 | |  | 11. InC1 | 4.6 | 1 | 1 |
| 12. Ach2 | 4.7 | 1 | 1 |  | 12. CN4 | 4.3 | 1 | 1 | |  | 12. InC2 | 4.8 | 1 | 1 |

For each item, the number corresponds to the main scale item, and the abbreviated name corresponds to the names presented in Tables 3-6.

CVI: Content Validity Index, CVR: Content Validity Ratio, CA: Care appraisal, InG: Information-giving, Com: Communication, Con: Continuity, Ach: Antenatal checks, CQ: Care quality, CN: Care needs, APC: Adequacy of postnatal care, HPC: Health professional communication, InC: Individualised care

n for impact score = 10 women, n for CVI & CVR = 16 experts

**Supplementary Table S2** Pearson’s r correlations between EMC scale and sub-scale scores (n=324)

| EMC scales & subscales | PR | CA | InG | Com | Con | ACh | LB | CQ | CN | PN | APC | HPC | InC |
| --- | --- | --- | --- | --- | --- | --- | --- | --- | --- | --- | --- | --- | --- |
| EMC-pregnancy (PR) | 1.00 | 0.92 | 0.89 | 0.92 | 0.56 | 0.72 | 0.38 | 0.35 | 0.38 | 0.34 | 0.28 | 0.32 | 0.33 |
| Care appraisal (CA) |  | 1.00 | 0.79 | 0.79 | 0.44 | 0.59 | 0.35 | 0.34 | 0.34 | 0.34 | 0.28 | 0.31 | 0.34 |
| Information (InG) |  |  | 1.00 | 0.81 | 0.37 | 0.53 | 0.37 | 0.33 | 0.39 | 0.32 | 0.24 | 0.31 | 0.32 |
| Communication (Com) |  |  |  | 1.00 | 0.37 | 0.55 | 0.38 | 0.35 | 0.37 | 0.31 | 0.25 | 0.31 | 0.30 |
| Continuity (Con) |  |  |  |  | 1.00 | 0.45 | **0.07** | **0.04** | **0.09** | **0.07** | **0.09** | **0.05** | **0.07** |
| Antenatal checks (ACh) |  |  |  |  |  | 1.00 | 0.29 | 0.28 | 0.28 | 0.29 | 0.27 | 0.26 | 0.25 |
| EMC-Labour & birth (LB) |  |  |  |  |  |  | 1.00 | 0.96 | 0.95 | 0.65 | 0.58 | 0.57 | 0.63 |
| Care quality (CQ) |  |  |  |  |  |  |  | 1.00 | 0.82 | 0.63 | 0.56 | 0.54 | 0.62 |
| Care needs (CN) |  |  |  |  |  |  |  |  | 1.00 | 0.61 | 0.54 | 0.55 | 0.58 |
| EMC- postnatal (PN) |  |  |  |  |  |  |  |  |  | 1.00 | 0.88 | 0.93 | 0.92 |
| Adequacy of postnatal care (APC) | | | | |  |  |  |  |  |  | 1.00 | 0.71 | 0.71 |
| Health professional Communication (HPC) | | | | |  |  |  |  |  |  |  | 1.00 | 0.81 |
| Individualised care (InC) | | | | |  |  |  |  |  |  |  |  | 1.00 |

EMC: Experiences of maternity care

All P values are less than 0.001 except those in bold, which are greater than 0.05

**Supplementary Table S3** Pearson’s r correlations between EMC scale and sub-scales and participants’ age (n=324)

| EMC scale & subscales | r | P |
| --- | --- | --- |
| EMC-pregnancy | -0.03 | 0.53 |
| Care appraisal | -0.04 | 0.44 |
| Information-giving | 0.03 | 0.55 |
| Communication | -0.03 | 0.54 |
| Continuity | -0.10 | 0.70 |
| Antenatal checks | -0.03 | 0.60 |
| EMC-Labour & birth | -0.02 | 0.66 |
| Care quality | -0.02 | 0.66 |
| Care needs | -0.02 | 0.69 |
| EMC-postnatal | -0.04 | 0.47 |
| Adequacy of postnatal care | -0.07 | 0.23 |
| Health professional communication | -0.02 | 0.73 |
| Individualised care | -0.03 | 0.61 |

EMC: Experiences of maternity care

All P values greater than 0.05

**Supplementary Table S4** Cronbach’s alpha and Interclass Correlation Coefficient of each EMC scale and subscales (n=324)

| Scale/Subscale | no. of items | Alpha  (n = 216) | ICC (95% CI)  (n = 15) |
| --- | --- | --- | --- |
| EMC-Pregnancy | 12 | 0.91 | 0.88 (0.64-0.96) |
| Care appraisal | 3 | 0.76 | 0.95 (0.86-0.98) |
| Information giving | 2 | 0.81 | 0.69 (0.64-0.89) |
| communication | 3 | 0.77 | 0.87 (0.60-0.95) |
| Continuity | 2 | 0.78 | 0.99 (0.97-1.00) |
| Antenatal checks | 2 | 0.33 | 1.00 |
| EMC-Labour & birth | 12 | 0.92 | 0.93 (0.78-0.98) |
| Care quality | 7 | 0.87 | 0.91 (0.74-0.97) |
| Care needs | 5 | 0.86 | 0.93 (0.80-0.98) |
| EMC-Postnatal | 12 | 0.92 | 0.95 (0.88-0.98) |
| Adequacy of postnatal care | 4 | 0.86 | 0.98 (0.95-0.99) |
| Health professionals communication | 4 | 0.84 | 0.79 (0.37-0.93) |
| Individualised care | 4 | 0.77 | 0.93 (0.78-0.98) |

EMC: Experiences of maternity care

All P values greater than 0.05
